# Carboxymethyl cellulose‐polylactic acid particles for inhibiting anoikis and enhancing wound healing efficacy of human mesenchymal stem cells

**DOI:** 10.1002/btm2.70003

**Published:** 2025-02-12

**Authors:** Dong‐Hyun Lee, You Bin Lee, Hyun Su Park, Young‐Ju Jang, Youn Chul Kim, Suk Ho Bhang

**Affiliations:** ^1^ School of Chemical Engineering Sungkyunkwan University (SKKU) Suwon Gyeonggi‐do Republic of Korea

**Keywords:** anoikis, carboxymethyl cellulose, cell viability, polylactic acid, stem cell therapy

## Abstract

Adult human mesenchymal stem cells (hMSCs) injection into the wound site promotes angiogenesis and the wound‐closing process by secreting various growth and immune‐modulating factors. However, lower cell attachment sites and the hypoxic microenvironment in the wound site limit their viability and engraftment rate, leading to programmed cell death, anoikis. We synthesized carboxymethyl cellulose‐coated polylactic acid (CMC‐PLA) particles to prevent anoikis by providing an attachable surface for hMSCs. In vitro experiments demonstrated enhanced viability and secretion of growth factors by hMSCs under severely hypoxic microenvironments, when CMC‐PLA particles provided attachment surfaces, compared to controls. Furthermore, in vivo experiments showed that CMC‐PLA particles injected with hMSCs improved collagen synthesis and wound closure more than those of the control groups. These findings suggest that CMC‐PLA particles effectively enhance the therapeutic potential of hMSCs by providing a supportive microenvironment, promoting cell survival, proliferation, and angiogenesis, thereby offering a promising approach for advanced wound healing therapies.

**Translational Impact Statement**

In this study, we confirmed that carboxymethyl cellulose‐coated polylactic acid (CMC‐PLA) is a highly effective biomaterial for stem cell therapy, offering new opportunities for advanced tissue repair and regeneration strategy. The CMC‐PLA could improve the therapeutic outcomes of stem cell‐based wound treatments by enhancing cell viability, angiogenesis, and tissue regeneration. These results suggest the possibility of CMC‐PLA as more effective and durable healing solutions, especially in complex wounds.

## 1 INTRODUCTION

Mesenchymal stem cells (MSCs) secrete various paracrine factors and possess immunomodulatory properties, making them suitable for treating a wide range of wound conditions.^1^ Despite these advantages, direct injection of MSCs into wound sites presents several challenges.^2^ One major issue is anoikis, a form of cell death that occurs when cells fail to adhere to the surrounding substrate.^3^ In an abnormally hypoxic microenvironment of the wound area, poor cell adhesion can lead to cell death or inappropriate differentiation, thereby lowering the therapeutic efficacy of the treatment.^4^ To overcome these obstacles, polylactic acid (PLA)‐based scaffolds have been studied for cell delivery in wound healing applications. PLA is known for its biocompatibility and capacity to promote collagen synthesis, angiogenesis, and cellular activity, and can be applied for wound healing.^5^ However, PLA's limited capacity for cell adhesion due to its hydrophobicity poses a challenge when applied to stem cell therapies.^5^ To overcome this limitation, the PLA surface can be modified by introducing nanostructures that improve its cell‐binding capacity to enhance cell attachment and proliferation. For example, techniques such as electrospinning, plasma treatment, and laser ablation can be used to create nanostructured surfaces that mimic the natural extracellular matrix.^6^, ^7^ These nanostructures increase the surface roughness of the PLA scaffold, providing more binding sites for cell adhesion molecules and promoting better integration of stem cells into the scaffold.^8^ Moreover, the use of bioactive coatings or functionalization of nanostructured PLA surfaces with bioactive molecules (such as peptides, proteins, or growth factors) can enhance cell attachment, proliferation, and differentiation.^9^ However, these existing PLA modification methods require complex chemical processes, which can hinder their applications in the medical and cosmetic fields.

Carboxymethylcellulose (CMC) might offer a solution to address these issues. As a biocompatible and biodegradable polymer, CMC can be used in conjunction with stem cell therapy to provide a supportive matrix for cell adhesion.^10^, ^11^ The hydrophilic properties of CMC attract and retain moisture, creating an optimal environment for cell growth and differentiation, which are important for wound healing.^12^ Additionally, the surface of CMC can act as a physical scaffold, supporting stem cells and helping them to remain at the wound site.^13^, ^14^ Therefore, CMC might enhance cell proliferation and suppress anoikis by facilitating cell attachment, thereby improving the overall success of stem cell‐based treatments. Additionally, CMC, which is simpler and more biocompatible, can overcome the need for complex chemical processes associated with existing PLA modifications, thus facilitating their medical and cosmetic applications. Therefore, to overcome the limitations of PLA, we propose the use of CMC to enhance PLA's cell‐adhesive properties. Previous studies explored various materials to improve stem cell delivery and survival at wound sites. However, the use of a combination of CMC and PLA for enhanced stem cell therapy has not been extensively studied. This new method utilizes the advantages of both the materials, overcoming the limitations of their individual use.

In this study, we hypothesize that the combination of CMC and PLA can synergistically enhance stem cell adhesion and proliferation, thereby overcoming the limitations of PLA alone. This approach is expected to improve therapeutic outcomes in stem cell‐based wound healing by integrating the hydrophilic properties of CMC with the biocompatibility and structural support of PLA. CMC‐PLA particles are expected to provide an optimal surface for stem cell adhesion, thereby preventing anoikis and promoting the secretion of angiogenic factors when injected into wound sites. This study aimed to confirm whether the combination of CMC and PLA could lead to improved therapeutic outcomes in stem cell‐based wound healing. Therefore, this study aims to validate the potential of CMC‐PLA as a novel platform for regenerative medicine, offering a promising method to address challenges in wound healing and stem cell therapy.

## 2 MATERIALS AND METHODS

### 2.1 Synthesis of PLA and CMC‐PLA

PLA was dissolved in dimethyl sulfoxide (DMSO, Sigma‐Aldrich, MA, USA) to prepare a 7% PLA solution. The prepared 7% PLA solution was sprayed onto cooled hexane. The interaction between the PLA solution and hexane resulted in the formation of PLA particles. The PLA particles formed were dissolved in aqueous NaCl solution to remove DMSO. The washed PLA particles were mixed with the CMC solution. The mixture was freeze‐dried to obtain the final CMC‐PLA particles. To get optimum particle size, a sieving procedure was conducted using a 100 μm pore‐sized sieve.

### 2.2 Particles characterization

The morphologies of the PLA and CMC‐PLA particles were examined using a JSM7000F SEM microscope (JEOL, Japan), and Fourier‐transform infrared spectroscopy (FTIR) was obtained using an IRTracer‐100 spectrometer (Shimadzu, Japan). Zeta potential was determined using a Zetasizer Ultra Red label (Malvern, UK) after each particle was dispersed in phosphate‐buffered saline (PBS; Gibco BRL, NY, USA). The particle sizes were observed using an optical microscope (CKX53, Olympus, Japan), and the captured images were analyzed using Photoshop CC (Adobe Systems, CA, USA).

### 2.3 Cell culture

The human mesenchymal stem cells (hMSCs) were purchased from Lonza (Bazel, Switzerland). The hMSCs were cultured in Dulbecco's Modified Eagle's Medium (DMEM; Gibco BRL) supplemented with 10% (v/v) fetal bovine serum (FBS; Gibco BRL) and 1% (v/v) penicillin/streptomycin (PS; Gibco BRL). The cells were incubated at 37°C with 5% CO_2_ saturation. The cell culture medium was changed every 2 days. Cells within six passages were used for the experiments.

### 2.4 Cytotoxicity test

PLA and CMC‐PLA particles were suspended in the same cell culture medium (DMEM containing 10% FBS and 1% PS). The suspended particles were treated directly with 24‐well plate cultured hMSCs by various concentrations (1, 10, 100 μg/mL, and 1, 10 mg/mL) for 24 h to assess the cytotoxicity. A Cell Counting Kit (CCK)‐8 assay kit (Dojindo Molecular Technologies, Japan) was used to measure the cell viability at different concentrations of each particle. The CCK‐8 solution was added to each well and incubated for 2 h. Absorbance was measured at 450 nm using a microplate reader (Tecan, Switzerland). For the indirect toxicity test, the various concentrations (100 μg/mL, and 1, 10 mg/mL) of suspended particles were treated to cultured hMSCs by transwell for 72 h. Then, the CCK‐8 assay was proceeded with as the identical procedure as mentioned.

### 2.5 Cell adhesion analysis

To determine whether cells adhered to the particles under conditions mimicking the wound microenvironment, 2 × 10^4^ hMSCs were suspended in a poly‐hydroxyethyl methacrylate (p‐HEMA) coated 24‐well cell plate (to ensure a non‐adherent cell culture environment) containing 1 mg/mL of each particle. For the p‐HEMA coating, 1 mL of 2% p‐HEMA solution was embedded in a 24‐well cell plate for 24 h; after that, each well was washed twice by PBS. Thereafter, cells were incubated at 1% O_2_ saturation in serum‐free DMEM for 72 h.^15^ The CCK‐8 solution was added to each well and incubated for 2 h under normal incubation conditions. After incubation, collected medium was filtered by a 40 μm cell strainer (Corning, NY, USA), and absorbance was measured at 450 nm using a microplate reader (Tecan). For visualization, 1,1′‐dioctadecyl‐3,3,3′,3′‐tetramethylindocarbocyanine perchlorate (DiI, Sigma‐Aldrich) stained hMSCs were cultured with suspended particles.

### 2.6 Terminal deoxynucleotidyl transferase dUTP nick end labeling assay

To evaluate the apoptotic activity of cells after indirect particle treatment for 72 h, the cells were fixed with 4% (v/v) paraformaldehyde for 1 h. To evaluate the apoptotic activity of each group, the fixed cells were subjected to a terminal deoxynucleotidyl transferase dUTP nick end labeling (TUNEL) assay using an ApopTag1 fluorescent in situ apoptosis detection kit (Millipore, MA, USA) according to the manufacturer's instructions. After 40, 6‐diamidino‐2‐phenylin‐dole (DAPI, Vector Laboratories, CA, USA) staining, the cells were imaged using fluorescence microscopy (Leica).

### 2.7 Quantitative reverse transcription‐polymerase chain reaction

The quantitative reverse transcription‐polymerase chain reaction (qRT‐PCR) was used to quantify the relative gene expression levels of human Bcl‐2, BAX, Beclin, PCNA, Ki67, and mouse β‐actin, CD31, alpha‐smooth muscle actin (α‐SMA), and transforming growth factor beta‐1 (TGFβ‐1). Human‐specific gene primers were used for the in vitro hMSC analysis. Mouse gene primers were used for in vivo mouse skin tissue analysis. Total ribonucleic acid (RNA) was extracted from the samples using 1 mL Trizol reagent (Life Technologies, CA, USA) and 200 μL chloroform. The lysed samples were centrifuged at 12,000 rpm for 10 min at 4°C. The resulting RNA pellet was washed with 75% (v/v) ethanol in water and dried. After drying, samples were dissolved in RNase‐free water. For qRT‐PCR, the SsoAdvanceed™ Universal SYBR Green Supermix kit (Bio‐Rad, CA, USA) and CFX Connect™ real‐time PCR detection system (Bio‐Rad) were used.

### 2.8 Protein expression assay (western blot and enzyme‐linked immunosorbent assay)

The hMSCs cultured with each particle under hypoxic conditions were collected and lysed in radioimmunoprecipitation assay buffer (Rockland Immunochemicals Inc., PA, USA) that contained a protease inhibitor cocktail (Sigma‐Aldrich, P8340). After centrifugation at 10,000 × *g* for 10 min, the supernatant was used as the protein extract. Protein concentrations were determined using a bicinchoninic acid assay (Pierce Biotechnology, IL, USA). The same proteins from each sample were mixed with sample buffer, loaded, and subjected to sodium dodecyl sulfate‐polyacrylamide gel electrophoresis (SDS‐PAGE) using a 10% (v/v) resolving gel. The proteins separated by SDS‐PAGE were transferred to immune‐blot polyvinylidene fluoride membranes (Bio‐Rad) and probed with antibodies against glyceraldehyde 3‐phosphate dehydrogenase (GAPDH, Abcam, ab 9485, 1:2000, UK), caspase3 (Cell Signaling Technology, CS9662, 1:1000, MA, USA), Integrin α‐V (Abcam, ab179475, 1:1000), Akt (Cell Signaling Technology, CS4691, 1:1000), p‐Akt (Cell Signaling Technology, Cs4691, 1:1000), and collagen type 1 (Col I, Abcam, ab34710, 1:1000) for 4°C overnight. The membranes were then incubated with horseradish peroxidase‐conjugated secondary antibody (R&D Systems), HAF017 for GAPDH, caspase3, Integrin α‐V, Akt, p‐Akt, and Col I for 1 h at room temperature. The blots were then developed in a dark room. Luminescence was recorded using an x‐ray blue film (Agfa HealthCare NV, Belgium). Bands were imaged using Photoshop CC (Adobe Systems). The original uncropped western blotting images are included in Figure S1. To assess the amount of protein secretion, cell supernatants were collected using a 40 μm cell strainer (Corning) 72 h after incubating the cells with each particle under hypoxic, non‐adherent conditions. Cytokine concentrations were measured using enzyme‐linked immunosorbent assay (ELISA) kits for human vascular endothelial growth factor (VEGF; R&D Systems, USA) and hepatocyte growth factor (HGF; R&D Systems), according to the manufacturer's protocol.^16^

### 2.9 Angiogenesis and tube formation assay

Human angiogenesis (ARY007, R&D Systems) was conducted using the manufacturer's protocol to analyze the expression of angiogenesis‐related cytokines in hMSCs cultured with each type of particle. Tube formation was assessed using an angiogenesis assay kit (ab204726, Abcam), according to the manufacturer's instructions. Human umbilical vein endothelial cells (HUVECs) were seeded onto an extracellular matrix gel (2 × 10^4^ cells/well) in 100 μL of HUVEC culture medium and conditioned medium (CM) from cells incubated with each particle (3:1, v/v). and incubated for 4 h at 37°C. Following incubation, HUVECs were stained with a staining dye for 30 min at 37°C and observed under a fluorescence microscope (Leica).^17^

### 2.10 Cell scratch migration assay

Keratinocyte migration assays were performed in six‐well plates. Briefly, 1 × 10^5^ keratinocytes were added to each six‐well and scraped at 24 h after seeding. The CM from cells incubated with each particle was collected to treat each group. The number of cells that had migrated into the scraped area was calculated using brightfield microscopy connected to an optical microscope (Olympus CKX53). The cell migration area in the captured images was calculated using Photoshop CC software (Adobe Systems).

### 2.11 Skin wound‐closing model

Athymic mice (6 weeks old, female, 20–25 g body weight; Orient Bio Inc., Korea) were anesthetized via an intraperitoneal injection of xylazine (10 mg/kg) and ketamine (100 mg/kg) diluted in normal saline solution. The mid‐dorsal area of the skin of each mouse was incised to create a full‐thickness skin wound (2.0 × 2.0 cm^2^).^18^, ^19^, ^20^ After skin removal, eight 6–0 sutures (AILEE Co., Busan, Korea) were placed at the border of each wound to prevent wound collapse due to skin contracture. hMSCs and particles were subcutaneously injected at the edges of the wound (eight injections, 25 μL per site). All groups were treated with Tegaderm™ (3M Health Care, MN, USA), which is used as a common dressing material. All procedures, including skin removal, suturing, injection, and dressing, were completed within 30 min before the anesthesia wore off. Tegaderm skin dressings, with isoflurane inhalation for anesthesia, were replaced at 3, 7, and 10 days after inducing wound modeling. Wound closing was observed at 14 days. Wound closure was calculated as the percentage of the initial wound area ([wound area at time]/[initial wound area] × 100). All samples were collected in an identical manner to allow for an accurate comparison of wound closure among the different groups. All tissues from the dorsal wound area were retrieved for analysis to compare wound healing processes in each group. All animal treatments and experimental procedures were approved by the Institutional Animal Care and Use Committee of the Sung Kyun Kwan University (SKKU; SKKUIACUC2023‐06‐17‐2).

### 2.12 Histology and immunohistochemistry

Tissues retrieved from athymic mice were subjected to histological examinations. Skin tissue samples were embedded in the optimal cutting temperature compound (O.C.T. compound, Tissue‐Plus®; Scigen, NY, USA), frozen, and cut into 10 μm sections at −20°C. Overall tissue regeneration was evaluated by hematoxylin and eosin (H&E) and observed under a light microscope (CKX53, Olympus). Sections were subjected to immunofluorescence staining using an anti‐laminin antibody (Abcam, ab11575, 1:200), anti‐involucrin antibody (Abcam, ab53112, 1:100), and fluorescein isothiocyanate‐conjugated secondary antibody (Jackson ImmunoResearch Laboratories, PA, USA, 1:50) to visualize protein expression and wound repair. The sections were counter‐stained with DAPI (Vector Laboratories) and examined under a fluorescence microscope (Leica).

### 2.13 Statistical analysis

All data are presented as mean ± standard deviation (SD). The statistical analysis was performed using GraphPad Prism (GraphPad Software, USA). To determine statistical significance, an unpaired Student's *t*‐test was performed to compare two experimental groups, and an ordinary one‐way analysis of variance (ANOVA) cell proliferation‐related gene was performed for three or four experimental groups. Statistical significance was considered when the *p*‐value was less than 0.05, 0.01, 0.005, and 0.001.

## 3 RESULTS

### 3.1 Characterization of synthesized PLA and CMC‐PLA particles

**FIGURE 1:**
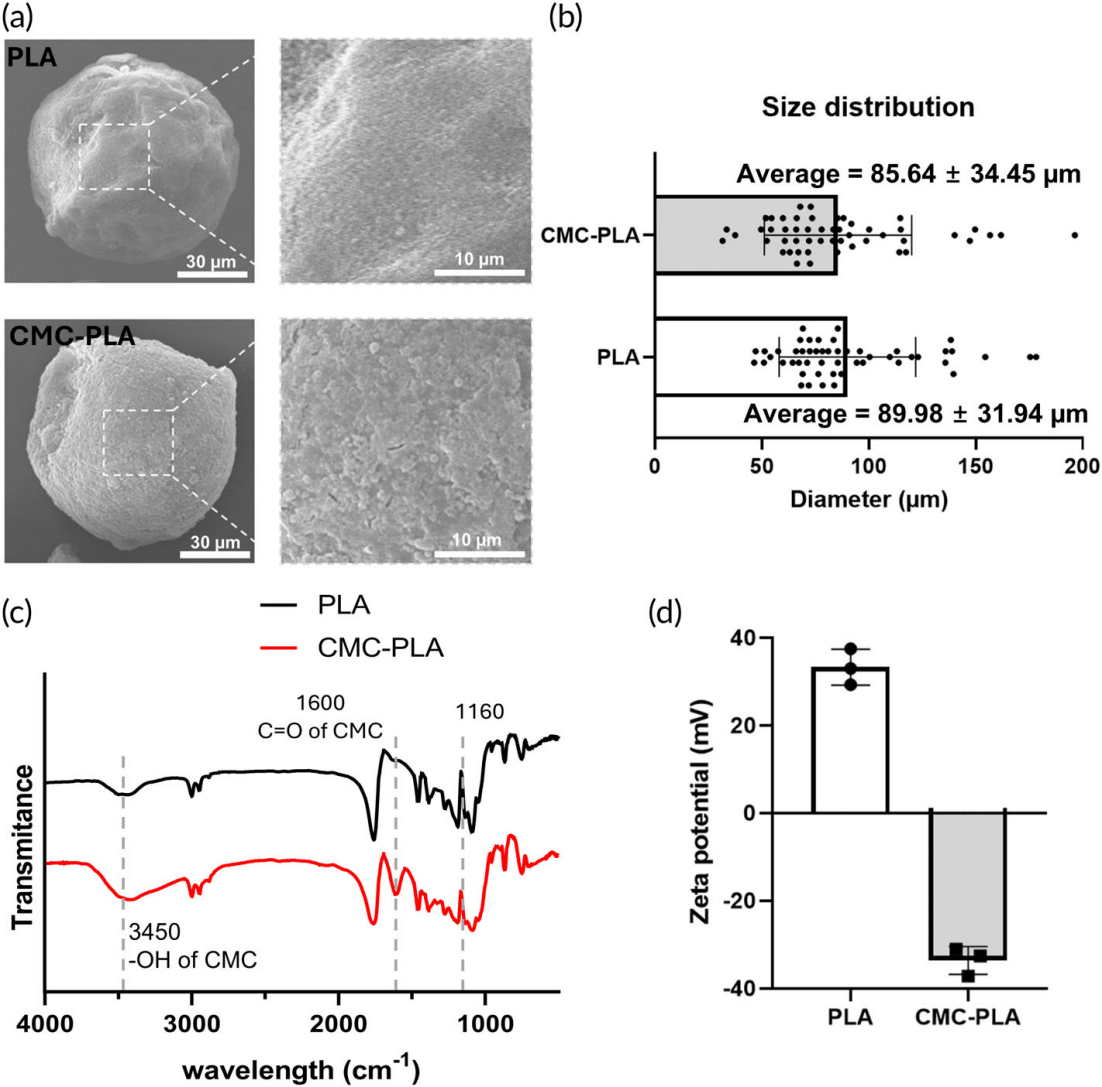
Characterization of polylactic acid (PLA) and carboxymethyl cellulose‐coated polylactic acid (CMC‐PLA) particles. (a) Representative scanning electron microscope images of each particle (scale bar = 30 μm [left], scale bar = 10 μm [right]), (b) size distribution (*n* = 50), (c) Fourier‐transform infrared spectroscopy spectra, and (d) zeta potential of PLA and CMC‐PLA particles.

We prepared PLA by spraying the PLA solution into cooled hexane, and the mixture of the prepared PLA and the dissolved CMC solution was freeze‐dried to obtain the final CMC‐PLA particles. Both final particles were sieved to obtain similar size distributions. The particle properties of the PLA and CMC‐PLA used in this study are shown in Figure 1. The surface topography of each particle was analyzed using a scanning electron microscope (SEM). The SEM images showed that both particle types are spherical. PLA particles showed a smoother surface; CMC‐PLA particles showed a rougher and more pitted surface than PLA particles (Figure 1a).^21^ The size distribution of each particle type was determined using an optical microscope. The average diameter of PLA particles was 85.64 ± 34.45 μm and CMC‐PLA particles was 89.98 ± 31.94 μm (Figure 1b). Micro‐particles with sufficient surface area to support cell adhesion and spreading are advantageous for promoting cell attachment. In this study, the micro‐particle size was approximately 85–90 μm, which was effective in facilitating wound healing.^22^ Furthermore, excessively large particle can lower the injectability into the skin dermal layer, highlighting the importance of optimizing the particle size.^23^ The size was determined for the optimum attachment of cells to these particles without affecting the cell‐binding ability and injectability to wound sites.^22^, ^23^ FTIR spectra of each particle showed different peak bands at 1160, 1600, and 3450 cm^−1^ (Figure 1c). The peak change at 1160 cm^−1^ indicated alterations in the ester linkage of PLA owing to the interactions between PLA and CMC.^24^ In addition, the band peak difference at 1600 and 3450 cm^−1^ is due to the C=O and hydroxyl groups of CMCs, indicating their incorporation into the micro‐particles.^25^, ^26^ Average zeta potential of PLA and CMC‐PLA particles was, respectively, 33.27 and −33.63 mV (Figure 1d).

### 3.2 Cytotoxicity of synthesized PLA and CMC‐PLA particles

**FIGURE 2:**
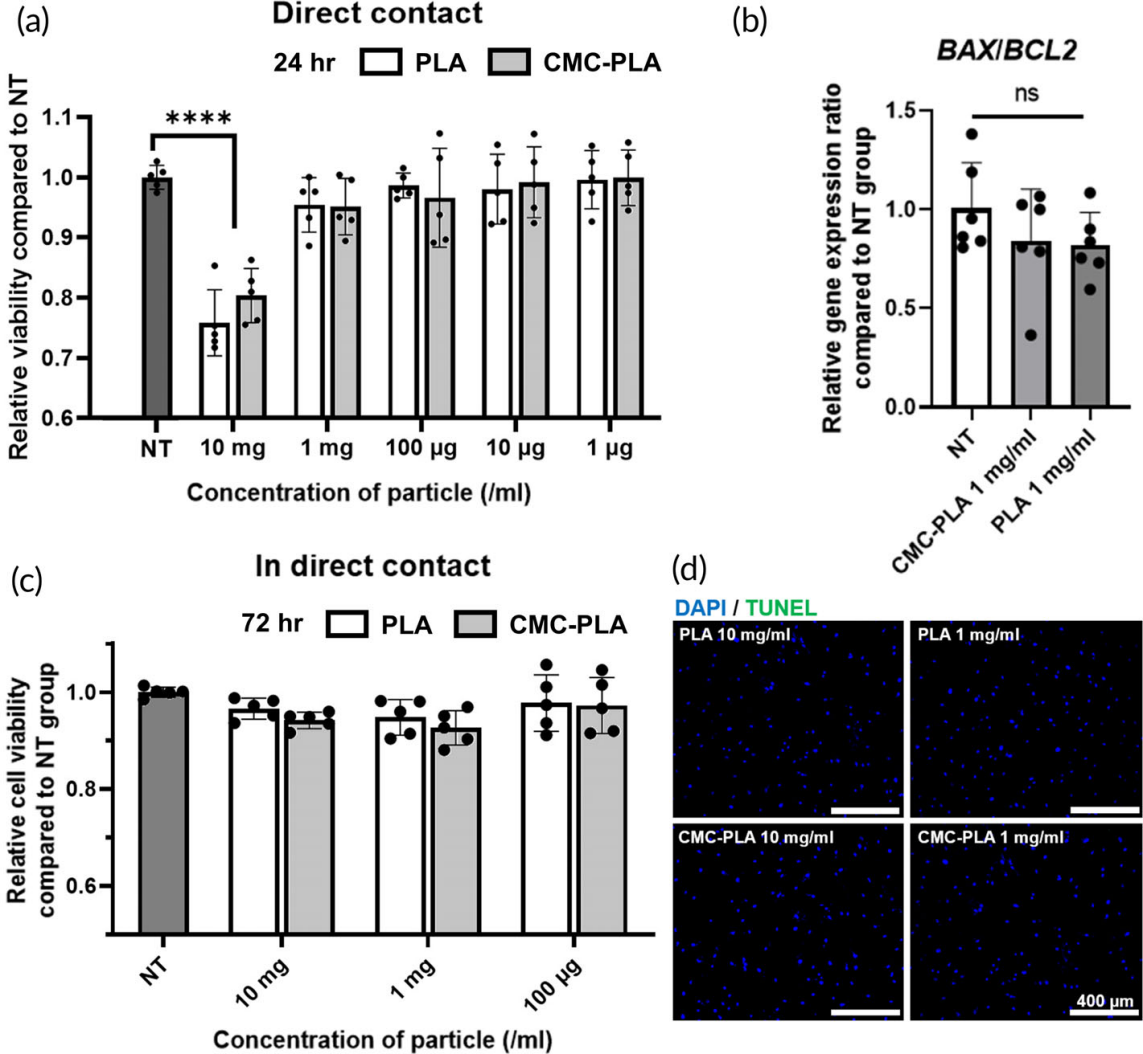
In vitro cytotoxicity test of synthesized polylactic acid (PLA) and carboxymethyl cellulose‐coated polylactic acid (CMC‐PLA) particles on human mesenchymal stem cells (hMSCs). (a) Cell viability after direct particle treatment with various concentrations (0, 10, and 1 mg/mL; 100, 10, and 1 μg/mL) of PLA and CMC‐PLA (*n* = 5, ****p < 0.001 compared to the no treatment (NT) group). (b) Relative apoptotic sensitivity (BAX/BCL2) of hMSCs after 1 mg/mL of each particle treatment (*n* = 6, ns, no significance). (c) Cell viability after in direct with particles for 72 h using transwell with various concentrations (0, 10, 1 mg/mL, and 100 μg/mL) of each particle (no statistical difference between the groups). (d) Representative images of terminal deoxynucleotidyl transferase dUTP nick end labeling‐stained (blue, nucleus; green, apoptotic cell) hMSCs, 72 h treating each particle with different concentration (scale bars = 400 μm). DAPI, 6‐diamidino‐2‐phenylin‐dole; TUNEL, terminal deoxynucleotidyl transferase dUTP nick end labeling.

The cytotoxicity of each particle at various concentrations was measured to determine the concentration of particles to be administered to the cells in the wound site. PLA and CMC‐PLA treatment directly on the surface of hMSCs did not exhibit cytotoxic effects at high concentrations, up to 1 mg/mL (Figure 2a). The PLA and CMC‐PLA 10 mg/mL concentration groups showed significant changes in cell viability compared to the no treatment group. However, the viability was maintained at >75% compared with the no treatment group, which is considered highly biocompatible.^27^ Through this result, PLA and CMC‐PLA at a concentration of 1 mg/mL were added together with cells for in vitro and in vivo experiments. Subsequently, the expression of apoptosis‐related genes *BCL‐2* and *BAX* was measured after treatment with 1 mg/mL of PLA and CMC‐PLA (Figure 2b). No significant differences were observed between the groups. The cytotoxicity of PLA and CMC‐PLA was assessed as an indirect treatment (Figure 2c). Each particle at different concentrations was treated on a transwell plate to investigate only the effects of the degraded substances. Indirect PLA and CMC‐PLA treatment of hMSCs did not exhibit cytotoxic effects, even at a concentration of 10 mg/mL. This result was confirmed by the TUNEL assay (Figure 2d). No apoptotic signals were detected in the fluorescence images.

### 3.3 Inhibited anoikis‐mediated cell death of hMSCs using CMC‐PLA particles

**FIGURE 3:**
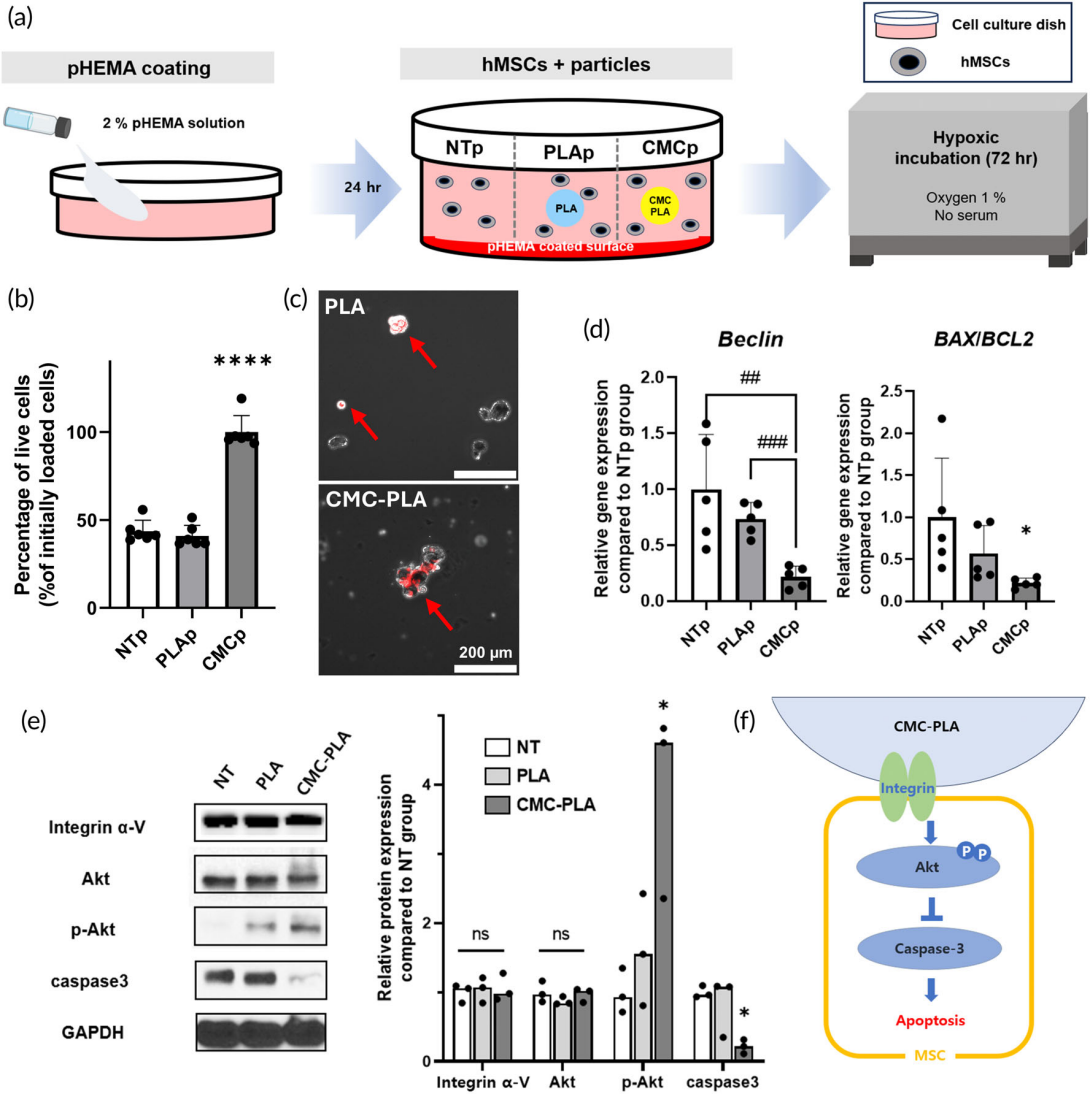
Inhibition of anoikis in human mesenchymal stem cells (hMSCs) using carboxymethyl cellulose‐coated polylactic acid (CMC‐PLA) particles under conditions mimicking the wound area. (a) hMSCs were incubated (72 h, 1% oxygen, without serum) with 1 mg/mL of each particle (PLAp and CMCp) or without particles (NTp) after coating the bottom surface of cell culture dishes with poly(2‐hydroxyethyl methacrylate (p‐HEMA). (b) Percentage of live hMSCs in each group after hypoxic incubation (*n* = 6). (c) Representative fluorescence images of DiI‐labeled hMSCs with each particle after hypoxic incubation (scale bar = 200 μm). (d) Relative cell detachment‐related gene (Beclin) expression and apoptotic activity (BAX/BCL2) of hMSCs after hypoxic incubation (*n* = 5). (e) Western blot analyses showing expression of anoikis‐related proteins in hMSCs with group (*n* = 3). (f) A schematic diagram of molecular mechanisms inhibiting anoikis in hMSC with CMC‐PLA. *p < 0.05 and ****p < 0.001 compared to other groups. ^##^ *p* < 0.01 and ^###^ *p* < 0.005 between two groups. ns, no significance.

Next, we assessed whether anoikis of the cells would be inhibited under conditions mimicking the wound environment by attachment to CMC‐PLA particles. The hMSCs were suspended in the wells of a p‐HEMA‐coated cell culture plate containing 1 mg/mL of each particle. The groups in which only hMSCs were suspended (NTp) and PLAs and CMC‐PLAs were added to hMSCs (PLAp and CMCp) were used in the experiment. The cells were incubated with 1% O_2_ in serum‐free DMEM for 72 h (Figure 3a). The relative cell viability in the CMCp group was twice that of other groups (Figure 3b). Additionally, in low serum, hypoxia condition, CMCp group also showed higher attachment compared to PLAp group (Figure S2). Fluorescence images of DiI (red signal)‐labeled hMSCs and each particle showed that cells incubated with PLA particles gathered, whereas cells incubated with CMC‐PLA particles were attached to the particles (Figure 3c). After 72 h of incubation, the gene expression data of the cell detachment‐ and apoptosis‐related genes, *Beclin* and *BAX/BCL‐2*, also exhibited the lowest expression levels in the CMCp group (Figure 3d),^28^ indicating that hMSCs were highly attached to the surface of the CMC‐PLA particles, resulting in less anoikis (cell detachment‐related cell death) than in the other groups (Figures 3b,c and S3). Based on the increased attachment of hMSCs to CMC‐PLA under non‐adherent hypoxic conditions, we investigated the effects of cell attachment to external particles and how this attachment decreased anoikis. Western blot analyses were performed to compare the expression levels of integrin alpha V, Akt, p‐Akt, caspase3, and GAPDH among the NTp, PLAp, and CMCp groups (Figure 3e). The results indicated no significant differences in the protein expression levels of integrin alpha V and Akt between groups. However, the expression level of p‐Akt in the CMCp group was more than fourfold higher than that in the NT and PLA groups. Concurrently, the expression level of caspase3 was more than four times lower in the CMCp group. The significant increase in p‐Akt expression indicates the activation of the cell survival pathway, and the noticeable decrease in caspase3 expression suggests the suppression of apoptosis.^29^, ^30^ Therefore, we confirmed that CMC‐PLA particles provided hMSCs with stable adhesion and enhanced their survival in harsh environments, such as the wound sites, via the Akt mechanism (Figure 3f).

### 3.4 Enhanced cellular migration and angiogenic ability of hMSCs with CMC‐PLA particles

**FIGURE 4:**
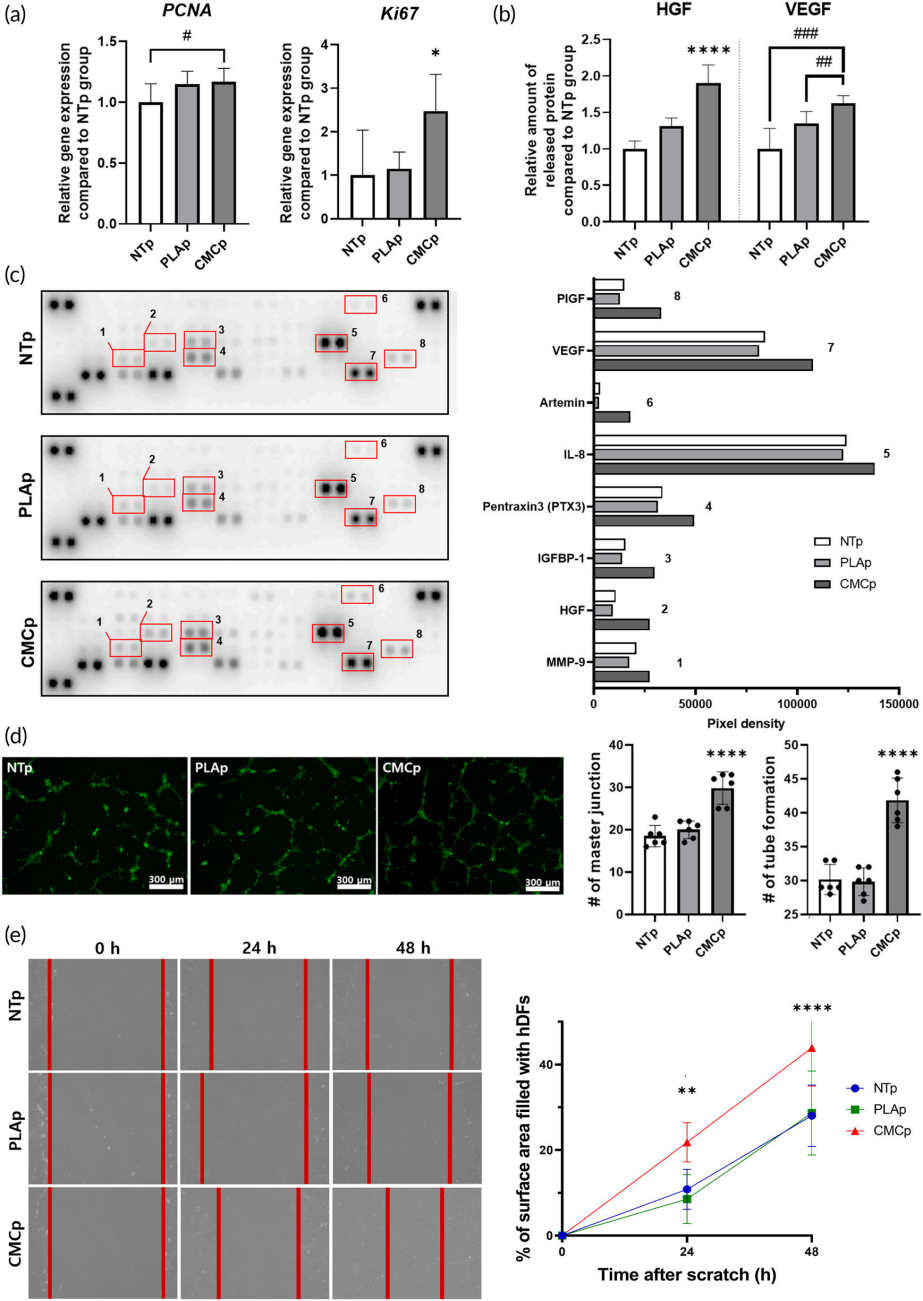
Enhanced angiogenic and wound healing effect of human mesenchymal stem cells (hMSCs) cultured with carboxymethyl cellulose‐coated polylactic acid (CMC‐PLA). (a) Relative expression of cell proliferation‐related genes in hMSCs after 72 h under hypoxic conditions with each particle (PLAp and CMCp) or without particles (NTp) (*n* = 5). (b) Relative wound healing‐related factors were secreted in the conditioned medium from hMSCs (72 h) of each group, as evaluated by enzyme‐linked immunosorbent assay (*n* = 5). (c) Expression of angiogenesis‐related proteins (*n* = 1), (d) human umbilical vein endothelial cells tube formation assay (scale bar = 300 μm, *n* = 6), and (e) scratch wound coverage assay (*n* = 4) results using condition medium of each group. *p < 0.05, **p < 0.01, and ****p < 0.001 compared to other groups. ^#^ *p* < 0.05, ^##^ *p* < 0.01, and ^###^ *p* < 0.005 between two groups. HGF, hepatocyte growth factor; PIGF, placenta growth factor; VEGF, vascular endothelial growth factor.

We examined whether the proliferative and angiogenic properties of hMSCs could increase when the cells were attached to CMC‐PLA particles under conditions mimicking the wound environment, as hypothesized. We investigated a series of proliferation‐related genes expression (Figure 4a) and angiogenesis‐related proteins secretion (Figure 4b) in each group. Figure 4a shows that hMSCs attached to CMC‐PLA expressed upregulated *PCNA* and *Ki67* compared with the other groups. In addition, as shown in Figure 4b, the secretion of angiogenesis‐related growth factors, HGF and VEGF, was increased 1.9‐ and 1.6‐fold, respectively, compared to the NTp group. Based on these results, an angiogenesis array was performed with CM collected from the hMSCs in each group to determine whether the secreted growth factors released from the cells would accelerate the wound healing process and stimulate blood vessel formation (Figure 4c,d). The proangiogenic proteins that showed differences between the groups are plotted in the graph (Figure 4c). A slight increase in the protein secretion of matrix metalloproteinase‐9 (MMP‐9), which is known to be involved in angiogenesis,^31^ was observed, and increased HGF and insulin‐like growth factor binding protein 1 (IGFBP‐1)^32^ secretion suggested improved cell growth and survival in the CMCp group. The elevated level of pentraxin3 that can indicate a proangiogenic microenvironment was confirmed. The angiogenic cytokine, interleukin‐8 (IL‐8), which stimulates angiogenesis, was also observed.^33^ Furthermore, artemin and placenta growth factor (PIGF), that are related to the promotion of vascular growth and development, were also investigated.^34^ Additionally, the CMCp group showed an increased secretion of VEGF, which is one of the most significant angiogenic growth factors.^35^

To compare the effects of angiogenic growth factors in each group, we treated HUVECs with CM from the NTp, PLAp, and CMCp groups and assessed tube formation (Figure 4d). The CMCp group exhibited a denser tubular network structure than those of the PLAp and NTp groups. The numbers of master junctions and tubes formed in the CMCp group exceeded those in the other groups. Specifically, the number of master junctions in the CMCp group was more than 1.5 times higher than that in the NTp group, and the number of tubes formed was approximately 1.4 times higher than that in the NTp group. These findings indicated that the CMCp group showed changes in the secretion of proangiogenic growth factors, as shown in Figure 4c, thereby stimulating new vessel formation.^36^

To determine the migration properties of hMSCs treated with each type of particle under conditions that mimic the wound environment, we performed a wound scratch assay. Figure 4e shows that keratinocytes in the CM of the CMCp group exhibited increased cell migration compared to those in the CM of the NTp and PLAp groups. In addition, the wound coverage rate graph shows that the keratinocyte migration area of the CMCp group was quantitatively enhanced by more than twice that of the NTp and PLAp groups (Figure 4e).^37^

### 3.5 Accelerated wound healing effect of injected hMSCs with CMC‐PLA particles

**FIGURE 5:**
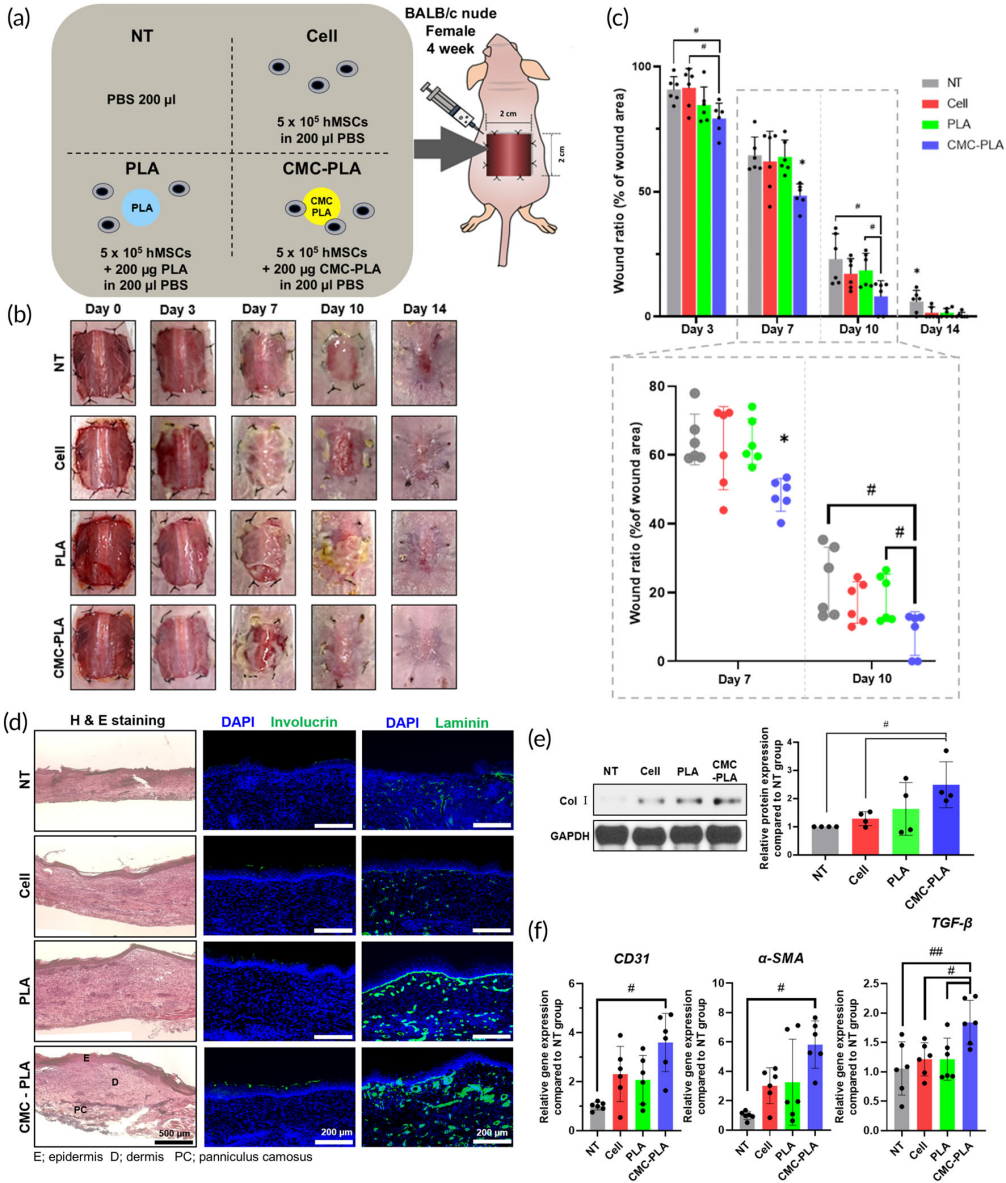
Enhanced in vivo wound healing efficacy of injected human mesenchymal stem cells (hMSC) with carboxymethyl cellulose‐coated polylactic acid (CMC‐PLA). (a) Schematics showing a mouse wound‐closing model using hMSC with different groups. (b) Representative images of wound at 0, 3, 7, 10, and 14 days after various treatments. (c) Quantification of wound closure at each day (right, *n* = 6). (d) Representative hematoxylin and eosin (H&E)‐stained images (left) and immunohistochemistry for involucrin (green; middle) or laminin (green; right) with 6‐diamidino‐2‐phenylin‐dole (DAPI) (blue) staining in skin wound at 14 days after the treatments. Relative expression of (e) Col I protein, (f) CD31, α‐SMA, and TGF‐β genes in the wound region at 14 days after the treatments (e) *n* = 4, (f) *n* = 6). *p < 0.05 compared to other groups. ^#^ *p* < 0.05 and ^##^ *p* < 0.01 between two groups. PBS, phosphate‐buffered saline.

To assess the therapeutic effects, we injected hMSCs with each particle into the skin wounds of BALB/c nude mice on Day 0 after skin wound modeling (Figure 5a). Figure 5b,c shows a comparison of the wound healing process among the untreated group (NT), injected hMSCs group (Cell), injected hMSC with PLA particles group (PLA), and with CMC‐PLA particles group (CMC‐PLA). Representative photographs of the mice at 0, 3, 7, 10, and 14 days after treatment are shown in Figure 5b. Wound closure was represented as a percentage of the original wound area. As Figure 5c shows, the skin wound area significantly decreased in the CMC‐PLA group (48.3%) compared to that in the NT (64.5%), Cell (62.0%), and PLA (63.8%) groups on Day 7 after wound modeling. In addition, on Day 10, the skin wound area remarkably decreased in the CMC‐PLA group (8.1%) compared to that in the NT (23.0%), Cell (17.1%), and PLA (18.5%) groups. By Day 14, while all groups showed wound closure in the representative images, the wound closure rate was significantly accelerated in the CMC‐PLA group compared to that in the other groups. The representative H&E‐stained results indicated enhanced re‐epithelialization in the CMC‐PLA group, with the tissue structure closely resembling normal skin^38^ (Figure 5d, left). In addition, immunohistochemistry (IHC) staining showed that the expression of involucrin and laminin increased in the CMC‐PLA group (Figure 5d, middle and right).^39^ In addition, expression of Col I protein in the skin tissue of each group was identified, and the expression was highest in the CMC‐PLA group compared to that in the other groups (Figure 5e). Col I, a key structural protein of the extracellular matrix, promotes wound healing of wounds.^40^ We also assessed the expression of angiogenesis‐ and wound regeneration‐related gene (Figure 5f). Expression of *CD31* and *α‐SMA* was increased more than three‐ and fourfold compared to the NT group, respectively, which is known as expressed in the process of early angiogenesis.^41^ Expression of *TGF‐β1*, also increased statistically significantly compared to other groups, which is important for the overall process of wound healing and is known to stimulate various cells at the wound site.^42^ Therefore, the increase in protein and gene expression of these factors indicates that the wound healing efficiency was enhanced in skin wounds. Overall, our experimental results demonstrated that hMSCs injected with CMC‐PLA particles could significantly facilitate the wound healing process and improve the structural reconstruction of the skin tissue.

## 4 DISCUSSION

**SCHEME 1:**
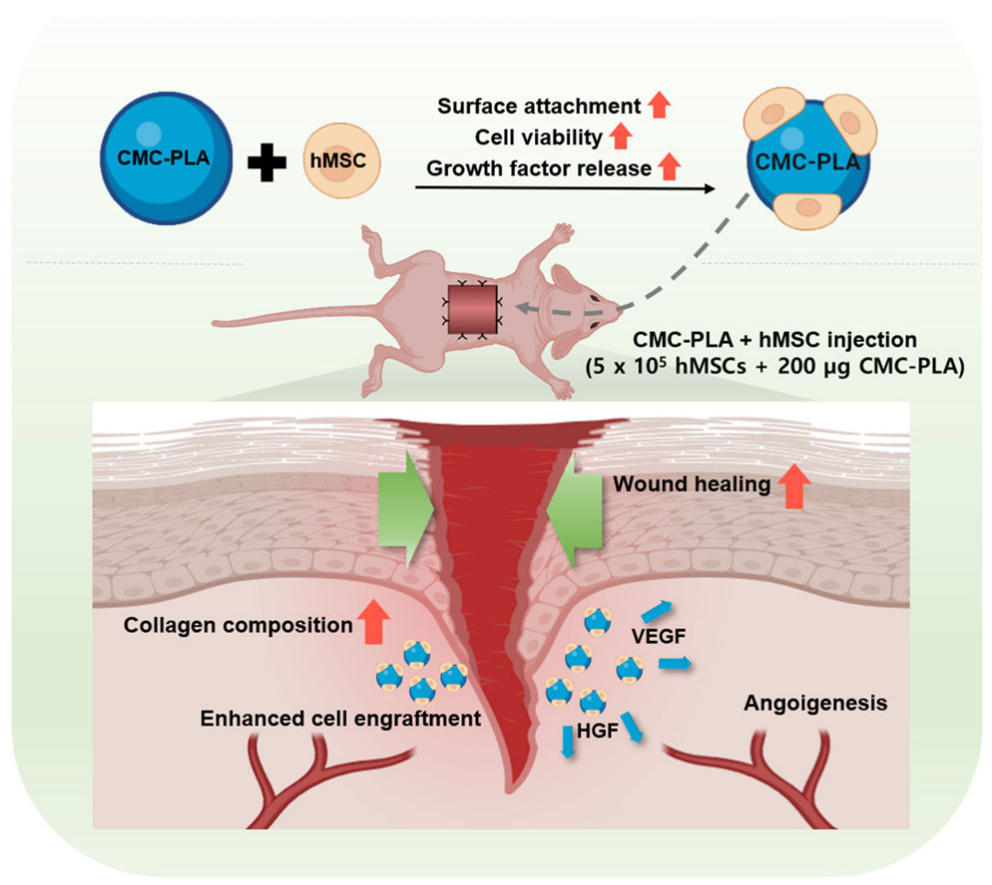
Schematics show the enhanced wound healing effect of human mesenchymal stem cells (hMSCs) with carboxymethyl cellulose‐coated polylactic acid particles (CMC‐PLA). Cell viability and growth factor secretion from the hMSCs were increased with CMC‐PLA particles. HGF, hepatocyte growth factor; VEGF, vascular endothelial growth factor.

Recently, stem cell therapy, particularly MSCs therapy, has begun to attract attention for wound healing owing to the regenerative properties of MSCs and the secretion of paracrine factors. However, challenges, such as anoikis, limit the efficacy of direct stem cell injection into wound sites.^43^ Previous studies on hydrogels and encapsulation have been conducted to overcome the limitations of conventional cell injection methods.^44^ Despite these efforts, further research in this field is required because of the limitations of previous studies.^45^, ^46^ To overcome these challenges, we propose a strategy using CMC in the form of biodegradable synthetic polymer (PLA) particles. Hydrogels have previously been applied for wound healing and were shown to significantly improve the therapeutic effect when administered together with CMC.^47^ Hydrogels combined with CMC have shown promising results, but hydrogels in general exhibit swelling properties that can potentially cause tissue damage.^48^, ^49^ Additionally, hydrogels tend to degrade more rapidly in the in vivo environment compared to synthetic polymer particles such as PLA, limiting their long‐term therapeutic effectiveness. To solve these problems, we decided to use CMC with PLA particles. By combining the cell‐adhesive property of CMC with the structural advantages of PLA, we developed CMC‐PLA particles that enhance cell proliferation and adhesion in the wound environment. This study aimed to demonstrate that CMC‐PLA particles significantly improve the efficiency of stem cell therapy for wound healing (Scheme 1).

The successful synthesis of the PLA and CMC‐PLA particles was confirmed by a combination of physical and chemical analyses (Figure 1). The representative SEM images illustrate that both types of particles had a spherical shape, with the CMC‐PLA particles exhibiting a rougher and more pitted surface than the smoother surface of the PLA particles. This morphological difference suggests the successful incorporation of CMC into the PLA particles, which enhanced the surface area and potentially improved the interaction of the particles with the biological environment (Figure 1a).^21^, ^50^ The size distribution analysis showed that both particle types had similar average diameters, approximately 85–90 μm, which is suitable for cellular attachment and injection into wound sites (Figure 1b).^22^ The FTIR spectra provided further evidence of CMC incorporation, with characteristic peaks at 1160, 1600, and 3450 cm^−1^ indicating the presence of CMC functional groups (Figure 1c).^24^ The zeta potential measurements demonstrated a significant shift from a positive value in the PLA particles to a negative value in the CMC‐PLA particles, confirming the alteration in the surface charge due to CMC integration (Figure 1d). The high magnitude of the zeta potential (more than ±30 mV) in both particles suggested that both types of particles are likely to be stable in suspension.^51^

Cytotoxicity assays revealed that both the PLA and CMC‐PLA particles exhibited high biocompatibility and cell viability at a concentration of 1 mg/mL. Additionally, cell viability remained above 75% even at higher concentrations. This indicated that the particles did not induce significant cytotoxic effects on hMSCs, making them suitable for biomedical applications (Figure 2a).^27^ The expression levels of the apoptosis‐related genes *BCL‐2* and *BAX* were not significantly different among the groups, further confirming the non‐toxic properties of the particles (Figure 2b). Additionally, the indirect treatment conditions assessed via transwell assays indicated that degraded substances from the particles also did not exhibit cytotoxic effects, ensuring the safety of these particles in long‐term applications (Figure 2c,d).

The ability of the CMC‐PLA particles to inhibit anoikis was demonstrated under conditions mimicking the wound environment (Figure 3a). hMSCs incubated with CMC‐PLA particles showed significantly higher cell viability than those in the other groups, indicating enhanced cell attachment and survival (Figure 3b). The fluorescence imaging and gene expression analyses further supported this result, showing that hMSCs adhered to CMC‐PLA particles more effectively, leading to the reduced expression of detachment and apoptosis‐related genes (Figure 3b,c).^28^ The western blot analysis revealed that the expression level of p‐Akt was significantly higher in the CMC‐PLA group, while caspase3 expression was notably lower (Figure 3e,f).^29^, ^30^ This suggests that the attachment of CMC‐PLA particles activates the Akt pathway, thereby enhancing cell survival and reducing apoptosis. These findings suggest the potential of CMC‐PLA particles to provide a stable adhesion surface for hMSCs, promoting cell survival in challenging environments such as wound sites.

The attachment of hMSCs to CMC‐PLA particles significantly enhanced their proliferation and angiogenic properties. The upregulation of proliferation‐related genes (*PCNA* and *Ki67*) and the increased secretion of angiogenesis‐related growth factors (HGF and VEGF) in the CMC‐PLA group indicate that these particles establish a helpful environment for cell growth and vascularization (Figure 4a,b).^52^, ^53^ The angiogenesis array and tube formation assays further demonstrated that the CM from the CMC‐PLA group stimulated endothelial cell tube formation more effectively than that from the other groups, suggesting that CMC‐PLA particles can enhance angiogenesis (Figure 4c,d). In addition, the secretion of proangiogenic proteins such as MMP‐9, IL‐8, and VEGF in the CMC‐PLA group indicated a potential angiogenic response, which is critical for effective wound healing.^54^ The wound scratch assay showed that hMSCs treated with CMC‐PLA particles exhibited significantly increased cell migration, further supporting the potential of these particles to promote wound healing by enhancing cell migration and angiogenesis (Figure 4e).^55^

In vivo experiments in BALB/c nude mouse models demonstrated the therapeutic potential of hMSCs with CMC‐PLA particles for accelerating wound healing (Figure 5a). The CMC‐PLA group showed a significantly reduced wound area and enhanced re‐epithelialization compared with the control and other treatment groups (Figure 5b,c). Histological analysis revealed that the CMC‐PLA group had tissue structures closely resembling those of normal skin, with increased expression of involucrin, laminin, and Col I, indicating improved structural reconstruction of the skin (Figure 5d,e). The gene expression analysis showed elevated levels of angiogenesis‐ and wound regeneration‐related genes (*CD31*, *α‐SMA*, and *TGF‐β1*) in the CMC‐PLA group, suggesting that these particles enhance the overall wound healing process by promoting angiogenesis and tissue regeneration.^56^ These findings demonstrate that CMC‐PLA particles not only support hMSC survival and proliferation but also significantly enhance their therapeutic efficacy in wound healing applications.^57^ Overall, these results demonstrate that CMC‐PLA not only supports the adhesion and survival of hMSCs but also enhances their angiogenic capacity, which could be highly beneficial for tissue engineering and regenerative medicine applications.

## 5 CONCLUSIONS

In this study, we demonstrated that CMC‐PLA provides an adhesive surface for stem cells, preventing cell anokis and promoting cell survival in harsh environments such as wound sites. hMSCs cultured with CMC‐PLA under non‐adherent hypoxic conditions showed higher viability, increased angiogenic factors, enhanced cell proliferation, and migration than control groups. Furthermore, hMSCs injected with CMC‐PLA in the mouse wound closure model showed significantly improved wound healing outcomes compared with the other groups. These results indicate that CMC‐PLA effectively enhances the therapeutic potential of stem cell treatments by providing a supportive microenvironment that promotes cell survival, proliferation, and angiogenesis. Although the result of this study is promising, further studies are required to improve the production and application of CMC‐PLAs. Future studies should focus on assessing the long‐term effects and safety of CMC‐PLA might be required. Our findings confirmed that CMC‐PLA is a highly effective biomaterial for stem cell therapy and offers a promising approach for improving wound healing. The synergistic properties of CMC and PLA make this combined material a valuable tool in regenerative medicine and provide new opportunities for the development of advanced therapeutic strategies for tissue repair and regeneration.

## AUTHOR CONTRIBUTIONS

**Dong‐Hyun Lee:** Conceptualization; methodology; data curation; visualization; writing – original draft; investigation; formal analysis; resources. **You Bin Lee:** Conceptualization; methodology; investigation; writing – original draft; formal analysis; software. **Hyun Su Park:** Methodology; investigation. **Young‐Ju Jang:** Formal analysis; methodology; validation. **Youn Chul Kim:** Project administration; funding acquisition; writing – review and editing. **Suk Ho Bhang:** Writing – review and editing; supervision; project administration; conceptualization; funding acquisition; resources.

## FUNDING INFORMATION

This research was supported by a Korean Fund for Regenerative Medicine (KFRM) grant funded by the Korea government (the Ministry of Science and ICT, the Ministry of Health & Welfare; project number: 21A0102L1‐12), the KIST Institutional Program (Project No. 2E32351, 2E32350 “Development of cellular bloc platform technology”, Korea), and the Materials/Parts Technology Development Program (1415187291, Development of composite formulation with a sustained release(gene) for the treatment of companion animal sarcopenia), Yangyoung Foundation of Samyang Corporation and the Technology Innovation Program (Project No. 20013794, Center for Composite Materials and Concurrent Design) funded by the Ministry of Trade, Industry & Energy (MOTIE, Korea).

## CONFLICT OF INTEREST STATEMENT

The authors declare no potential conflicts of interest with respect to the research, authorship, and/or publication of this article.

## Supporting information


**Figure S1.** The original uncropped western blotting images corresponding to Figures 3e and 5e (GAPDH, caspase3, Integrin α‐V, Akt, p‐Akt, and Col I). The strips marked with red boxes are the representative groups used in the article.
**Figure S2**. Relative cell adhesion of hMSCs in each group after 72 h of hypoxic (O_2_ 1%) incubation under 5% serum conditions (*n* = 6). ***p < 0.005 between two groups.
**Figure S3**. Representative scanning electron microscope (SEM) images of (a) PLA and (b) CMC‐PLA particles cultured with hMSCs for 72 h under hypoxic condition without serum addition (scale bar = 50 μm). The black arrows indicate hMSCs attached to CMC‐PLA particles.

## Data Availability

The data that support the findings of this study are available from the corresponding author upon reasonable request.
