# Supplementary material for: Carboxymethyl cellulose‐polylactic acid particles for inhibiting anoikis and enhancing wound healing efficacy of human mesenchymal stem cells
Source: Bioeng Transl Med. 2025 Feb 12;10(4):e70003. doi: 10.1002/btm2.70003 (PMC12284428; doi:10.1002/btm2.70003)
Supplement: Supplementary file 1 — Figure S1. The original uncropped western blotting images corresponding to Figures 3e and 5e (GAPDH, caspase3, Integrin α‐V, Akt, p‐Akt, and Col I). The strips marked with red boxes are the representative groups used in the article. Figure S2. Relative cell adhesion of hMSCs in each group after 72 h of hypoxic (O2 1%) incubation under 5% serum conditions (n = 6). ***p < 0.005 between two groups. Figure S3. Representative scanning electron microscope (SEM) images of (a) PLA and (b) CMC‐PLA particles cultured with hMSCs for 72 h under hypoxic condition without serum addition (scale bar = 50 μm). The black arrows indicate hMSCs attached to CMC‐PLA particles. [file BTM2-10-e70003-s001.docx]

**Supporting Information**

**Carboxymethyl cellulose-polylactic acid particles for inhibiting anoikis and enhancing wound healing efficacy of human mesenchymal stem cells**

Dong-Hyun Lee^1,*^, You Bin Lee^1,*^, Hyun Su Park^1^, Young-Ju Jang^1^, Youn Chul Kim^1^, Suk Ho Bhang^1^

*^1^ School of Chemical Engineering, Sungkyunkwan University (SKKU), 2066 Seobu-ro, Jangan-gu, Suwon, Gyeonggi-do 16419, Republic of Korea*

**Co-First authors (*)**

These authors contributed equally to this manuscript

**Corresponding author**Suk Ho Bhang, Ph.D., School of Chemical Engineering, Sungkyunkwan University (SKKU), 2066 Seobu-ro, Jangan-gu, Suwon, Gyeonggi-do 16419, Republic of Korea, E-mail: [sukhobhang@skku.edu](mailto:sukhobhang@skku.edu)


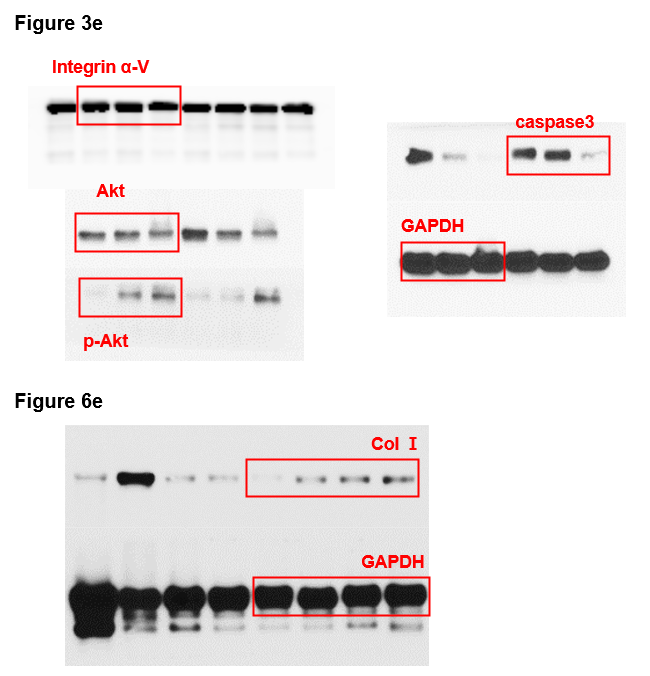


**Figure S1.** The original uncropped western blotting images corresponding to Figures 3e and 5e (GAPDH, caspase3, Integrin α-V, Akt, p-Akt, and Col I). The strips marked with red boxes are the representative groups used in the article.


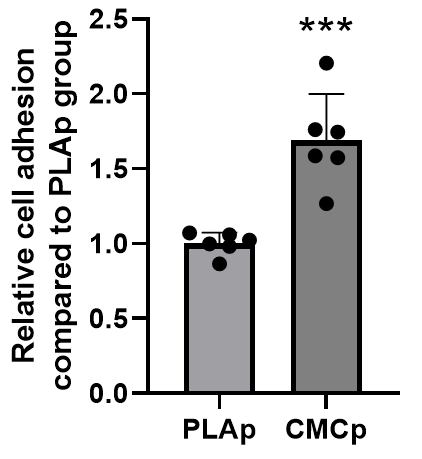


**Figure S2.** Relative cell adhesion of hMSCs in each group after 72 h of hypoxic (O_2_ 1%) incubation under 5% serum conditions (n = 6). *** p < 0.005 between two groups.

**
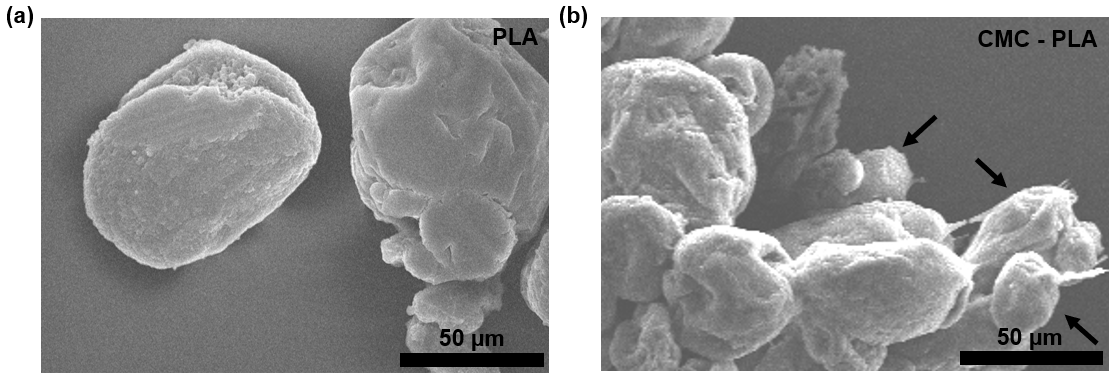
**

**Figure S3.** Representative scanning electron microscope (SEM) images of (a) PLA and (b) CMC-PLA particles cultured with hMSCs for 72 h under hypoxic condition without serum addition (scale bar = 50 µm). The black arrows indicate hMSCs attached to CMC-PLA particles.
